# Optimization and Testing of Hybrid 3D Printing Vitrimer Resins

**DOI:** 10.3390/polym14235102

**Published:** 2022-11-24

**Authors:** Jaime Casado, Osman Konuray, Gerard Benet, Xavier Fernández-Francos, José Maria Morancho, Xavier Ramis

**Affiliations:** Thermodynamics Lab, ETSEIB—UPC, Avda. Diagonal 647, 08028 Barcelona, Spain

**Keywords:** vitrimer, photoresin, hybrid polymers, acrylate, epoxy

## Abstract

The quality of photocure-based 3D printing greatly depends on the properties of the photoresin. There are still many challenges to be overcome at the material level before such additive manufacturing methods dominate the manufacturing industry. To contribute to this exciting re-search, an acrylate-epoxy hybrid and vitrimeric photoresin was studied to reveal the formulation parameters that could be leveraged to obtain improved processability, mechanical performance, and repairability/reprocessability. As the network becomes more lightly or densely crosslinked as a result of changing monomer compositions, or as its components are compatibilized to different extents by varying the types and loadings of the coupling agents, its thermomechanical, tensile, and vitrimeric behaviors are impacted. Using a particular formulation with a high concentration of dynamic β-hydroxyester linkages, samples are 3D printed and tested for repair and recyclability. When processed at sufficiently high temperatures, transesterification reactions are triggered, allowing for the full recovery of the tensile properties of the repaired or recycled materials, despite their inherently crosslinked structure.

## 1. Introduction

Conceived in the 1980s and initially intended merely for prototyping, additive manufacturing (or 3D printing) has garnered industrial and academic interest during the last decade. This is mainly due to the versatility and speed of processing that it entails, making it invaluable in an era driven by customized, small-scale, and make-to-order type industrial practices. There are excellent papers in the literature reviewing the different 3D printing technologies and their products for industries such as biotechnology, automotive and aerospace, sporting goods, and food [1,2,3,4,5,6,7,8,9,10].

Several thermoplastic- or thermoset-based polymer systems can be processed using different 3D printing methods. Apart from these conventional materials, a relatively new class of polymer materials, namely vitrimers, can also be used as building blocks for additive manufacturing. These materials combine the ease of processing of thermoplastics with the mechanical performance of thermosets [11,12,13,14,15]. This capability stems from their covalent but dynamic bonds that can reshuffle given appropriate stimuli, such as heat. The presence of such dynamic bonds, given sufficient temperature and time, permits even fully crosslinked polymer networks to disintegrate and allows for macroscale recycling through facile processes, such as by grinding and molding. The use of vitrimers in the realm of 3D-printing is somewhat new. Intended mostly for sterolithographic applications, such as digital light processing (DLP) printing, photosensitive resins are formulated from vitrimeric monomers that feature the aforementioned dynamic bonds based on chemistries such as disulfides [16,17] or esters [18,19] that can undergo bond exchange reactions when heated. These exchange reactions enable the repair and reprocessing of printed parts, using simple welding or hot pressing procedures.

**Scheme 1:**
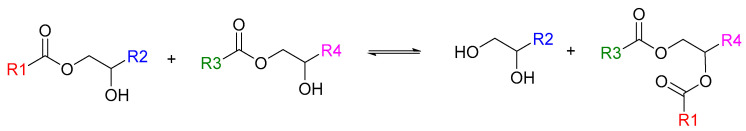
Transesterification between β-hydroxyesters.

In this work, we expose parameters that should be controlled in order to optimize an acrylate-epoxy hybrid and vitrimeric photoresin formulation so that a three-way balance is achieved between ease of (photo) processing, mechanical properties, and repairability/reprocessability. Starting from a vitrimeric photoresin that was characterized previously [20], we obtain an improved formulation by exploiting the leverage points revealed by our investigation. We then conduct mechanical tests on printed and repaired/recycled samples with this improved formulation. The acrylate-epoxy polymer contains a high concentration of β-hydroxyesters that can undergo transesterification when a suitable catalyst, such as a zinc salt, is used [21,22,23,24] (Scheme 1).

This enables the facile repair and reprocessing of the cured material. Furthermore, the hybrid formulation confers added versatility to the production process since the acrylate:epoxy composition can be changed so as to tailor the thermomechanical properties of the final material [20,25]. Moreover, this hybrid formulation can be cured sequentially, wherein a quick photocuring process is followed by thermal curing.

## 2. Materials and Methods

The acrylate part of the basic hybrid formulation consists of a 1:1 mixture (by weight) of glycerol 1,3-diglycerolate diacrylate (GLYDA) and poly(ethylene glycol) methacrylate (PEGMA). In the improved formulation optimized for 3D printing, a different methacrylate, namely 2-Phenoxyethyl methacrylate (FEMA), was used. The epoxy component of the basic formulation is a DGEBA-type epoxy resin (DG) with an epoxy equivalent weight of 187 g/ee. Other epoxides used in the study are neopentyl glycol diglycidyl ether (NG), bis(3,4.epoxycyclohexylmethyl) adipate (CE), trimethylolpropane triglycidyl ether (TGE), poly(propylene glycol) diglycidyl ether with an average molecular weight of 640 g/mol (PPGGE) or 380 g/mol (PPGGEs), and phloroglucinol triglycidyl ether (TFG). For epoxy-acid curing, glutaric acid (GLU) is used. To compatibilize the acrylate and epoxy parts, glycidyl methacrylate (GMA), which bears both acrylate and epoxide functionalities, is used as a coupling agent. In the basic formulation, the total mass of acrylates is the same as the mass of DG. Furthermore, the number of acrylate groups contributed by GMA is the same as the acrylate groups contributed by GLYDA. Lastly, GLU is added in stoichiometry with the total epoxides. As a photoinitiator, diphenyl(2,4,6-trimethylbenzoyl)phosphine oxide (TPO) was used. As a transesterification catalyst (to confer the vitrimer behavior), zinc acetoacetonate (Zn) was used. For further details about this formulation and the sample preparation procedure in general, refer to our previous paper [20]. All monomer materials, except for DG and TFG, were purchased from Sigma Aldrich (Madrid, Spain) and used without purification. DG was kindly supplied by Po.Int.Er Srl. (Valfenera, Italy). TFG was supplied by Specific Polymers (Castries, France).

The formulations were either photocured using a Vilber-Lourmat UV oven (Vilber Lourmat Sté., Collégien, France), inside hand-made transparent molds with polytetrafluoroethylene (PTFE) spacers to obtain rectangular samples, or 3D printed using an Asiga UV Max385 DLP printer (Asiga, Sydney, Australia). In the latter process, the layer thickness was 100 µm, and the wavelength of irradiation was 385 nm. Each layer received an irradiation of 87.5 mJ·cm^−2^, except the initial layer, which received 175 mJ·cm^−2^, to avoid cohesive failure between the build platform and the printed part. After that, the samples were thermally treated to carry out the epoxy-acid reaction at 120 °C for 12 h in a Memmert convection oven. Complete cure of materials was confirmed using a temperature-controlled Brucker Vertex 70 FTIR spectrometer equipped with an attenuated total reflection (ATR) accessory (GoldenGate™, Specac Ltd., Orpington, UK). The FTIR spectra obtained during the two curing steps are given in Figures S1 and S2 (supporting information). A final thermal post-treatment at 180 °C was carried out, also in the same oven, for an additional 4 h to ensure the equilibrium of transesterification reactions.

A Mettler DSC3+ calorimeter (Mettler-Toledo, Greifensee, Switzerland) was used to measure both the $T_{g}$ and the polymerization heats. Photocuring experiments were performed using a Hamamatsu LC5 light source (Hamamatsu Photonics K.K., Hamamatsu, Japan) equipped with a Hg-Xe mid-pressure lamp adapted to a Mettler DSC821 calorimeter. The UV light intensity was ~36 mW cm^−2^, measured at 365 nm using a radiometer (Hamamatsu Light Power Meter, Model C6080-03, Hamamatsu Photonics K.K., Hamamatsu, Japan). To quantify the storage stability, conversions were calculated using $x=1-\Delta h_{residual}/\Delta h_{total}$, where $\Delta h_{residual}$ is the heat evolved during the thermal cure after UV irradiation of a sample stored for a known duration of time, and $\Delta h_{total}$ is the total reaction heat of a freshly prepared and photocured sample.

The cured material $T_{g}$ was taken as the halfway point of the heat capacity step observed in a scan at 10 °C min^−1^, following the DIN 51007 standard method.

For thermomechanical analysis, a TA Instruments DMA Q800 device (TA Instruments, New Castle, DE, USA) was used. Prismatic samples with dimensions of 1.5 × 10 × 30 mm^3^ (thickness × width × length) were analyzed using a single cantilever clamp with a free length of 10 mm at a frequency of 1 Hz and an amplitude of 15 µm at 3 °C min^−1^ from −50 °C up to the rubbery state to measure the storage modulus, loss modulus, and tan δ. Stress relaxation experiments were performed on samples with dimensions of 1.5 × 10 × 20 mm^3^ using a 3-point bending configuration with a preload force of 0.01 N and a fixed strain of 1%. The flexural modulus was determined at 25 °C using a 3-point bending configuration with the same dimensions as used in the stress relaxation experiments, using the same preload force and a force ramp of 3 N/min.

Thermogravimetric analysis (TGA) was performed using a Mettler TGA/SDTA 851e/LF/1100 thermobalance (Mettler-Toledo, Greifensee, Switzerland). Fully cured samples were analyzed under isothermal conditions at 180 °C and under 50 cm^3^ min^−1^ of nitrogen purge.

The recycling of finely chopped samples was performed using a Specac Atlas manual 15T hydraulic hot-press (Specac Ltd., Orpington, UK) at a pressure of 12 MPa in an aluminum mold at 180 °C for 8 h.

Tensile tests on type-IV standard (ASTM D638-14) dog-bone samples were performed using an Instron 3366 Universal Testing Machine (Instron, Norwood, MA, USA) equipped with an extensometer. The test was performed at a constant displacement rate of 2 mm·min^−1^ until specimen failure. Young’s modulus was calculated for each sample using the linear regime of its stress–strain response.

The viscosity of a particular formulation, namely FEMA 3D 25 (vide infra), was measured with a Brookfield RST Rheometer (AMETEK Brookfield, Middleboro, MA, USA) with a cone/plate accessory using a shear rate of 10 to 1000 s^−1^ at 25 °C.

## 3. Results and Discussion

### 3.1. Effect of Acrylate to Epoxy Ratio

The acrylate:epoxy ratio affects the crosslinking density of the cured material, since the average monomer functionality is affected by a change in this ratio. The same is true for the extent of vitrimeric behavior, since the concentration of β-hydroxy ester groups changes when the acrylate:epoxy ratio is changed. The reference formulation, described in a previous work, contains GLYDA and PEGMA in a 1-to-1 weight ratio [20]. Throughout the text, this formulation is referred to as BASE 3D. Keeping this acrylate composition unaltered (GLYDA:PEGMA = 1:1), the amount of total acrylates w.r.t. epoxies was changed to obtain 5 alternate formulations. These are tabulated in Table 1, as 0.25, 0.5, 2, 3, and 4. The numbers represent the acrylate to DG weight ratios.

**Figure 1:**
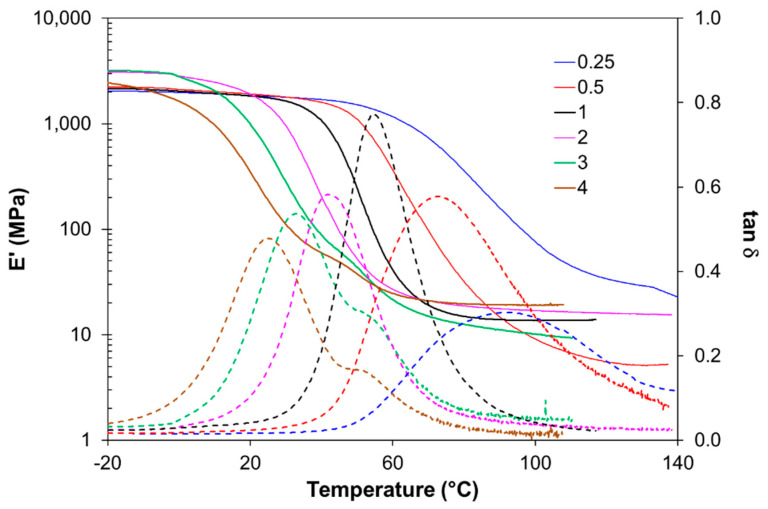
Storage moduli (*E’*) and tan delta curves of formulations with varying acrylate:epoxy ratios.

In Figure 1, the alpha relaxation of all formulations given in Table 1 can be seen. As expected, T_g_ increases as the epoxy-acid content increases because the network becomes more densely crosslinked. Besides, the aromatic rings coming from DG also increase the rigidity of the material. The plasticization effect of the aliphatic side chains from PEGMA is also reduced when the epoxy-acid content increases. Moreover, the lower the acrylate content, the wider the tan δ peaks because the networks are less homogeneous. On the other hand, acrylate-rich formulations exhibit a tan δ shoulder, suggesting an incompatibility between the acrylic and the epoxy-acid networks. This might be due to a decreased concentration of the coupling agent GMA in these formulations. Except for formulations 3 and 4, all are glassy solids at ambient temperature. The effect of thermal post treatment at 180 °C is more pronounced when the network is more epoxy rich. This is because the epoxy-rich networks contain an elevated concentration of β-hydroxy esters that undergo transesterifications. Whereas T_g_ of formulation-4 increases merely 2.4 °C upon post-treatment, the T_g_ of formulation-0.25 increases by 14 °C.

**Figure 2:**
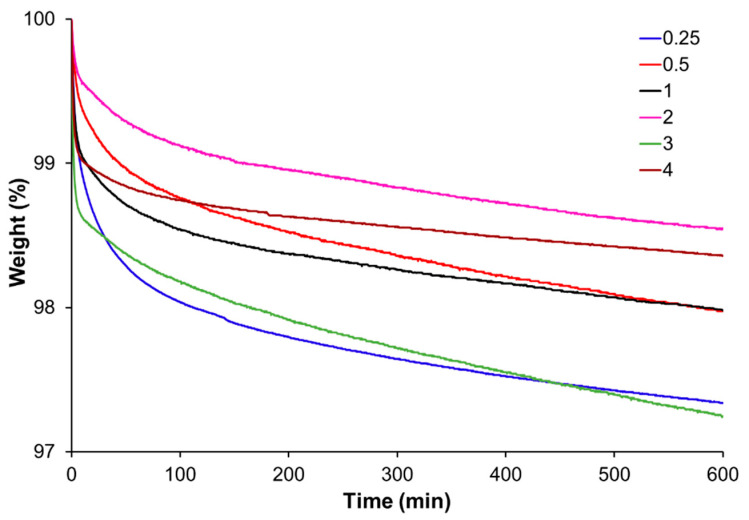
Remaining weight of formulations with varying acrylate:epoxy ratios treated at 180 °C.

As can be seen in Figure 2, the materials are thermally stable at the recycling temperature of 180 °C. Weight loss after 1 h and after 10 h of thermal treatment were recorded as less than 2% and 3%, respectively. This was also confirmed by solubility tests at 60 °C, in which a soluble fraction of 2% was found. This also confirms that the weight loss is predominantly due to the volatilization of low boiling point components, and not due to any thermal degradation.

**Figure 3:**
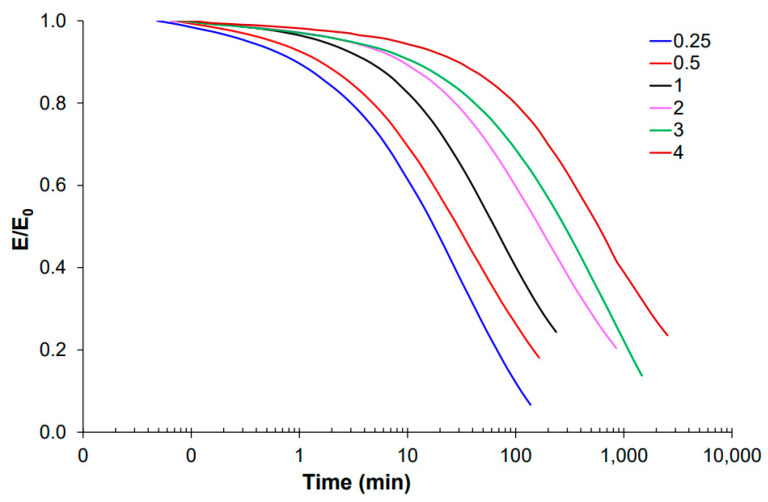
Stress relaxation at 180 °C of formulations with varied acrylate:epoxy ratios using the characteristic relaxation time.

As far as the vitrimeric performance is concerned, the stress relaxation behavior of epoxy rich formulations suggests that these materials would be more easily reprocessed compared to acrylate-rich ones (Figure 3). This is because the epoxy portion of the network contains a higher concentration of β-hydroxyesters and is less densely crosslinked than the acrylate portion. However, from the point of view of processing ability, acrylate-rich formulations have a lower viscosity and are therefore more suitable for the intended 3D-printing application. A trade-off exists between low viscosity and fast reprocessability, and in this sense, formulation 1 provides a good balance.

### 3.2. Effect of the Coupling Agent

To study the effect of GMA, the contents of GLYDA, PEGMA, and GLU were maintained as in formulation 1, while the GMA content was adjusted along with DG so that the total number of epoxy groups matched the number of acid groups of GLU. The TPO and Zn loadings were maintained at 2% and 6%, respectively. The tested variations are tabulated in Table 2. The formulation denoted as “0.8” in this table is the same formulation as the one denoted as “1” in Table 1.

**Figure 4:**
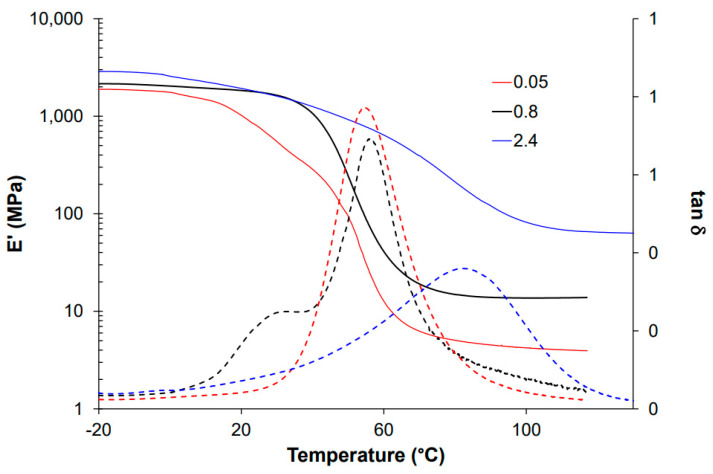
Alpha relaxations as influenced by the amount of coupling agent (GMA).

**Figure 5:**
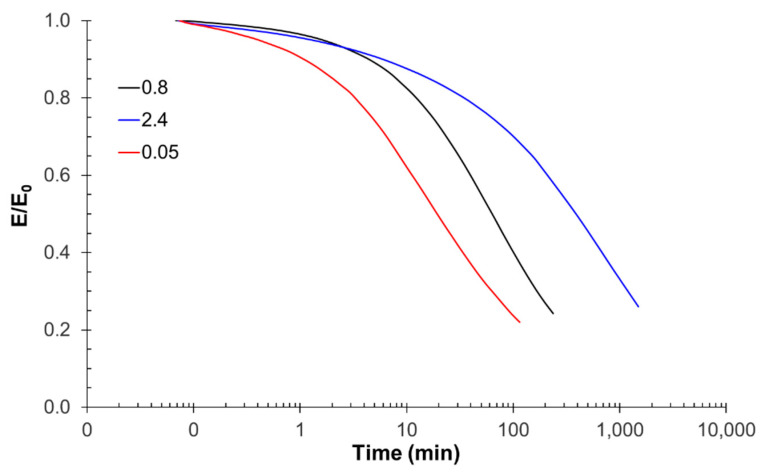
Stress relaxation as influenced by the amount of coupling agent (GMA).

As GMA content decreases, a bimodal tan δ results, suggesting a prejudicial effect on the network compatibility (Figure 4). On the other hand, as the GMA content increases, the tan δ peak is displaced to higher temperatures, and it widens considerably. As a matter of fact, the alpha-relaxation of the GMA-poor material starts earlier than it does in the base formulation containing 0.8 g GMA per gram of GLYDA. The increase in GMA, a small molecule bearing a difunctional methacrylate and an epoxy group, leads to a higher crosslinking density of the network once the epoxy group has reacted in the thermal curing stage, as evidenced by the increase in the storage moduli in the rubbery state. This is because the contribution of epoxy groups from DG is lessened, which would otherwise lead to a looser network structure. This also impacts the stress relaxation behavior, wherein an increase in GMA content slows down the relaxation rate at 180 °C, as can be seen in Figure 5. In fact, the β-hydroxyester concentration, provided by GLYDA and the epoxy-acid reaction, increases slightly with the GMA content. Therefore, this detrimental effect is mainly caused by the higher crosslinking density, significantly reducing the mobility of the network chains and impeding the dynamic bond exchange process. No appreciable change in thermal stability is observed when the GMA content is changed.

### 3.3. Effect of Epoxide Type

**Figure 6:**
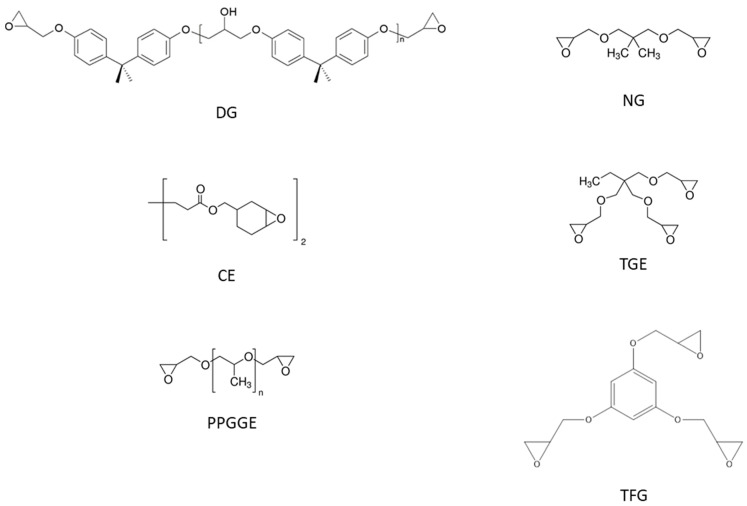
Different epoxides employed. For complete names, see Section 2.

To isolate the effect of epoxide type, the rest of the composition was maintained, while different epoxides were employed, replacing DG at the same molar ratio. The structures of the employed epoxides are given in Figure 6. PPGGE and PPGGEs are versions of the same monomer with different chain lengths, with equivalent weights of 640 and 380 g/mol, respectively. In Table 3, the compositions with different epoxides are given.

The choice of epoxide significantly impacts the final Tg, which ranges from −45 to 42 °C. Most materials are in the rubbery state at ambient temperature, except for the original formulation and when TFG is employed. The rigidity of the aromatic rings and the higher functionality of TFG, leading to a denser network structure, are responsible for this behavior. The materials also respond differently to thermal post-treatment, depending on the epoxide used (Table 4). For instance, formulations with TGE and CE exhibit sub-ambient Tg after curing at 120 °C, but after being post-treated at 180 °C, they become glassy solids at ambient conditions.

**Figure 7:**
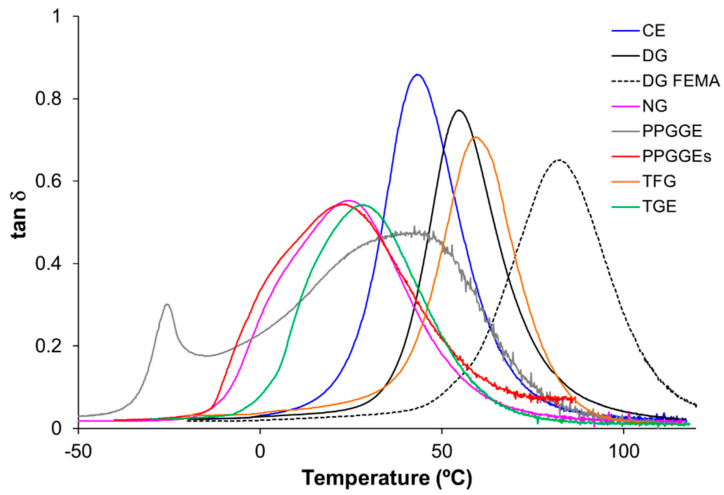
Tan δ curves of fully cured materials containing different epoxides. DG-FEMA formulation is the basic formulation in which PEGMA is replaced by FEMA.

As can be deduced from the tan δ curves given in Figure 7, network homogeneity also seems to be affected by the choice of epoxide, with some monomers, such as PPGGE, resulting in highly segregated networks. The long chain of PPGGE seems to disrupt the compatibility between the polyacrylate and epoxy-acid networks, the former network being rich in poly(propylene glycol) when PPGGE is used.

**Figure 8:**
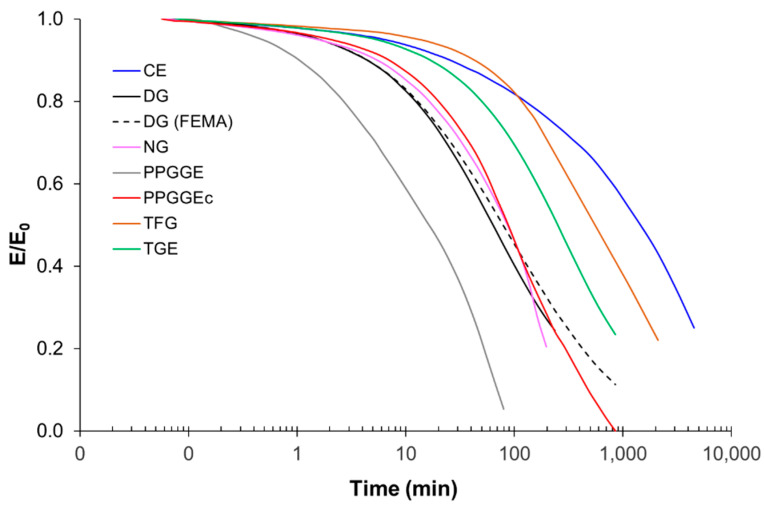
Stress relaxation of formulations containing different epoxides.

As per stress relaxation behavior, the variability is also high (Figure 8). Formulations with TGE, TFG, and CE exhibit very long characteristic relaxation times (i.e., τ*_0.37_). In particular, CE has a τ*_0.37_ of 3000 min, in stark contrast with basic formulation using DG, which has a τ*_0.37_ of only 114 min.

The fastest relaxing formulation is the one containing PPGGE, with a τ*_0.37_ of 30 min, but this material shows poor thermal stability. This is also true for the formulation with NG. The short-chain counterpart of PPGGE, namely PPGGEs, performs much better in this sense and exhibits stress relaxation behavior comparable to that of the basic formulation.

### 3.4. Optimization for 3D Printability

**Figure 9:**
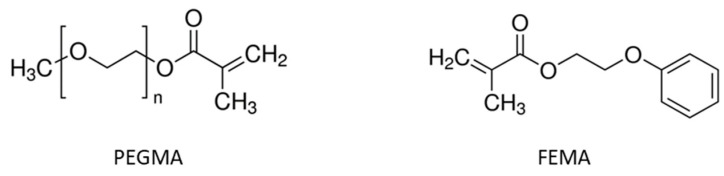
The monomethacrylates used in the study; PEGMA has an average molecular weight of 300 g/mol.

In this section, an optimal formulation will be presented that achieves a three-way balance between ease of processability (i.e., 3D printability), ultimate material T_g_, and reprocessability. Among the formulations presented in the previous section, the one containing PPGGEs is in the rubbery state at ambient temperature (T_g_ = −3 °C). If a glassy material is desired instead, the formulation can be modified in a number of ways, one of which is to employ PPGGEs as a co-monomer with DG, rather than replacing it altogether. A more drastic measure would be to replace the aliphatic PEGMA (Figure 9, left) with other methacrylates having more rigid backbones, such as FEMA (Figure 9, right).

Taking as a reference the basic formulation with DG, the result of this replacement is an increase in T_g_ from 42 to 64 °C; meanwhile, the thermal stability seems to be unaffected, and the stress relaxation behavior seems to be altered only slightly. The formulation with FEMA exhibits a τ*_0,37_. of 150 min, which is comparable to the formulation containing either DG or PPGGEs (See Figure 8).

As far as the epoxide composition is concerned, DG was partially replaced by PPGGEs to different extents, and a subset of these modified formulations was characterized (Table 5). These receive the suffix “3D”, since their low viscosities enabled their use as 3D printing resins. The number that follows is the weight percentage of PPGGEs based on total epoxide content.

**Figure 10:**
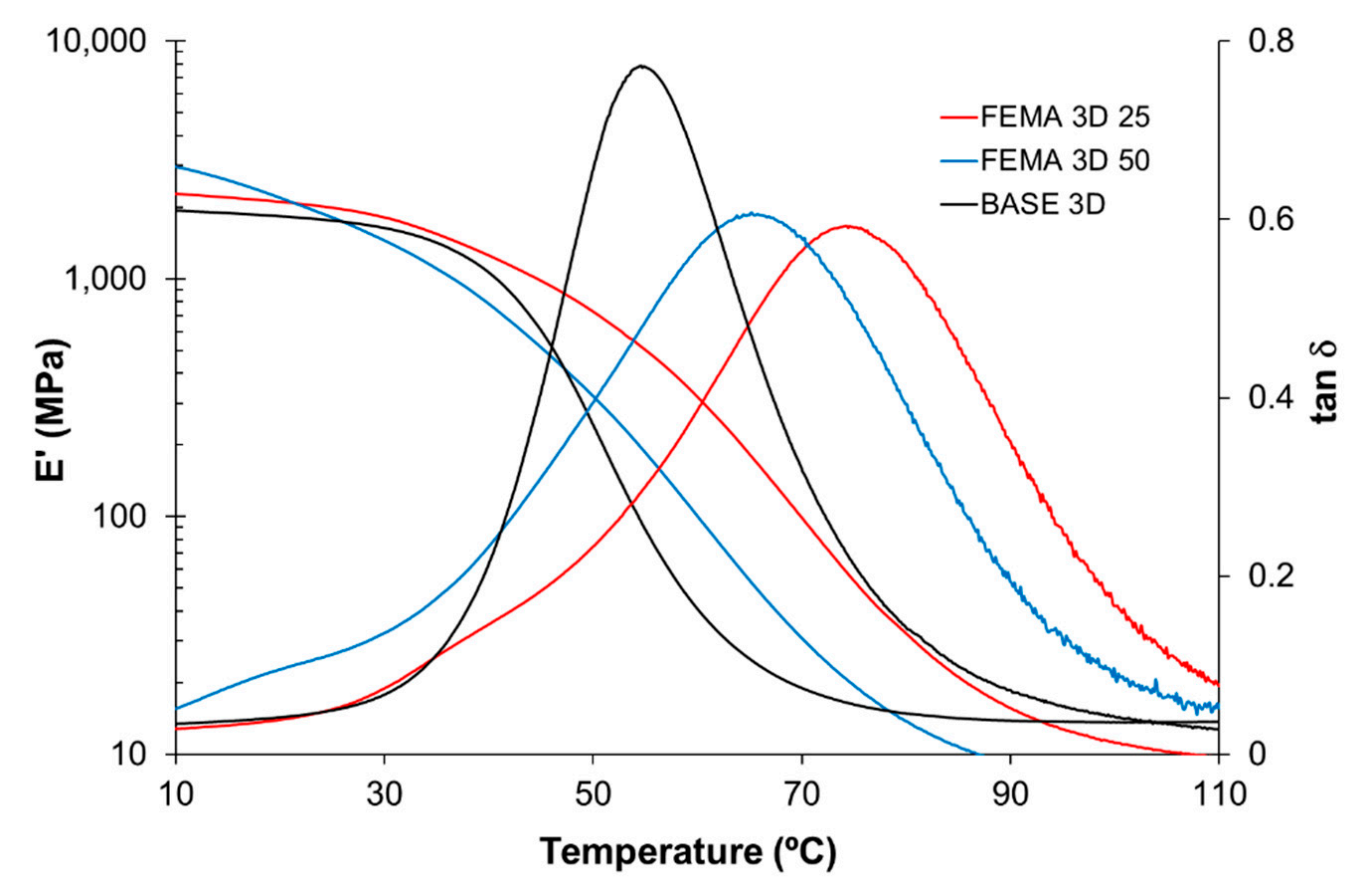
Storage moduli and tan δ of 3D printing formulations.

To compare the viscoelastic behavior of these modified formulations with that of the basic formulation (denoted as BASE 3D), DMA was performed. In Figure 10, tan *δ* and storage modulus *E’* curves of the 3 formulations can be compared. The alpha relaxation of *FEMA 3D 25* starts at the same temperature as for BASE 3D. Both are mostly glassy solids at ambient temperature. On the other hand, *FEMA 3D 50* starts to relax at the 10–20 °C temperature interval, suggesting that it may have poorer mechanical properties at ambient conditions.

**Figure 11:**
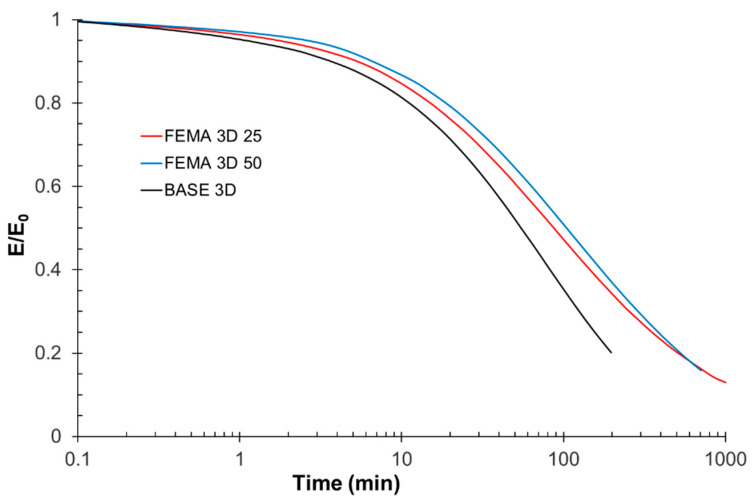
Stress relaxation at 180 °C of 3D printing formulations.

In terms of thermal stability, FEMA 3D 25 performed slightly worse than the basic formulation, but nevertheless retained 97% of its weight after 10 h at 180 °C, which is the reprocessing temperature. Its stress relaxation behavior is highly comparable to that of BASE 3D, as can be seen in Figure 11. The analysis of its Arrhenius stress relaxation kinetics is provided as supporting information (Figure S3 and Table S1). These results show that FEMA 3D 25 merits further testing as a 3D printing resin.

**Figure 12:**
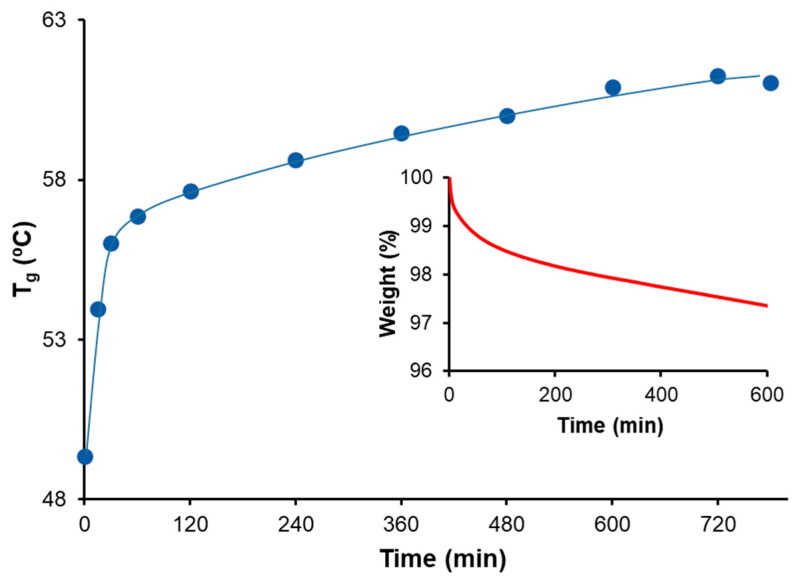
T_g_ increase in FEMA 3D 25 as a result of structural changes in the network during treatment at 180 °C. The TGA curve at this temperature is given as the inset.

Once heated to the post-treatment temperature of 180 °C, the T_g_ of the material starts to increase, as can be seen in Figure 12. This is due initially to the evaporation of volatiles already present in the sample and those that form secondarily due to transesterification reactions, leading to a rearrangement of the network structure. Possibly, this rearrangement produces volatile fragments which are later released, as suggested by the inset in Figure 12.

**Figure 13:**
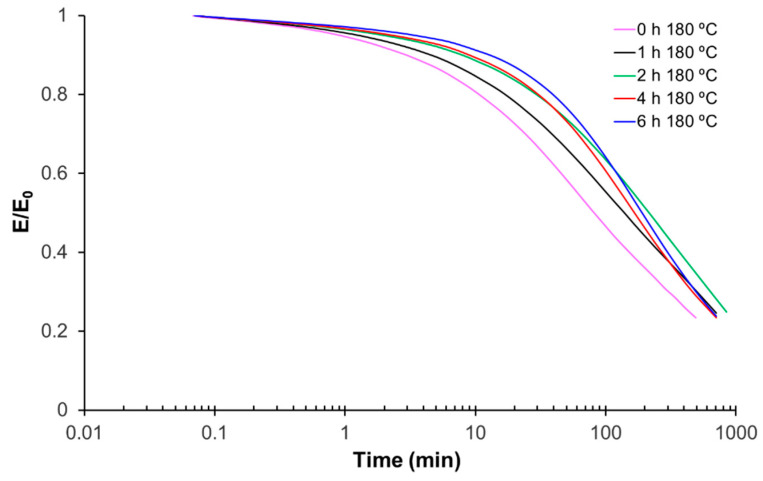
Stress relaxation behavior of FEMA 3D post-treated at 180 °C for different durations.

In fact, the evolution of T_g_ with time resembles the TGA curve for this material, which suggests that the increase in T_g_ is indeed due to the volatilization of light fragments. The higher initial rate of T_g_ increase might be due to originally detached moieties. This hypothesis is backed by solubility test results which indicated that material T_g_ increased after a 2.2% soluble fraction (by weight) was eliminated. The shape of the tan δ curve does not change during post-treatment, suggesting that network homogeneity is maintained (not shown). The duration of post-treatment has a similar effect on relaxation kinetics. Figure 13 provides the normalized relaxation moduli after different post-treatment durations. The impact of post-treatment is more pronounced at its 2 h onset, after which the stress relaxation behavior remains largely unaffected.

**Figure 14:**
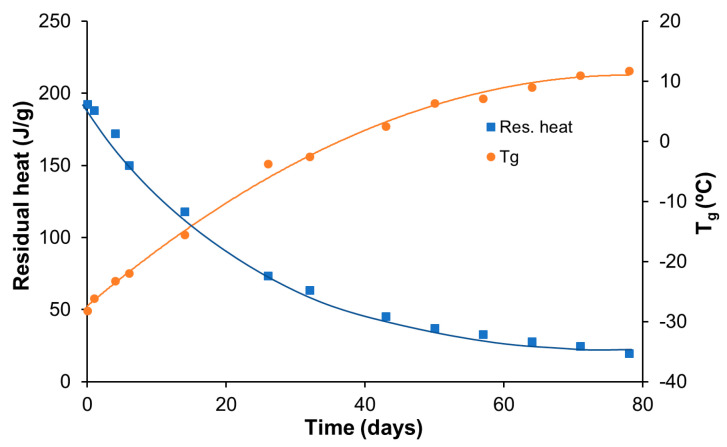
Prolonged storage stability monitoring of FEMA 3D 25.

As another preliminary test, the prolonged storage stability of FEMA 3D was tested. For this, samples of the liquid (uncured) formulation were kept at 30 °C in a thermostatic bath for a period of several months. The evolution of residual polymerization heats and T_g_, measured by DSC, are given in Figure 14. Despite the decrease in residual heat and the increase in T_g_ after 60 days, the resin remains liquid, albeit with a slightly increased viscosity. Tests with the DSC821 (UV) device revealed that no acrylate polymerization occurred during these 60 days, and the loss of stability is due to epoxy-acid reaction.

### 3.5. Printing, Repair, and Reprocessing

**Figure 15:**
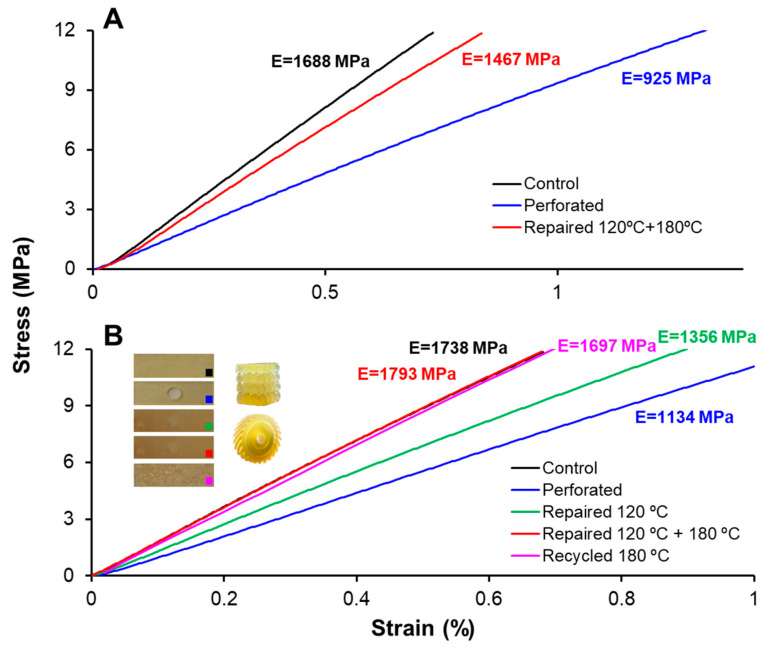
(**A**) The 3-point-bending stress–strain curves of pristine (black), perforated (blue), and repaired (red) FEMA 3D 25. (**B**) Stress–strain curves after repairing damaged samples of FEMA 3D 25 which were not post-treated at 180 °C during manufacture. Inset: Tensile testing samples, color coded to match the curves (left), a zeolite structure (top right), and a toothed gear (bottom right) printed with FEMA 3D 25.

DLP-3D printing is a simple additive manufacturing method that enables the production of parts with high resolution and high throughput. Complex functional pieces, such as those depicted in Figure 15B (inset), can be obtained with minimal preparation and good reproducibility.

For functional applications, it is crucial to characterize the mechanical performance of printed parts and also to know the extent to which damaged pieces can be repaired, taking advantage of their vitrimeric property. For this purpose, rectangular prisms were DLP-printed, some with a hole in their center (perforated), and then cured for 12 h at 120 °C and post-cured for 4 h at 180 °C. The reparation process consisted of filling the holes with the liquid formulation, followed by the dual UV+thermal curing at 120 °C procedure and the post-treatment at 180 °C for 4 h. As can be seen in Figure 15A, despite a significant improvement over the damaged sample, the modulus of the repaired sample falls short of the pristine one.

**Figure 16:**
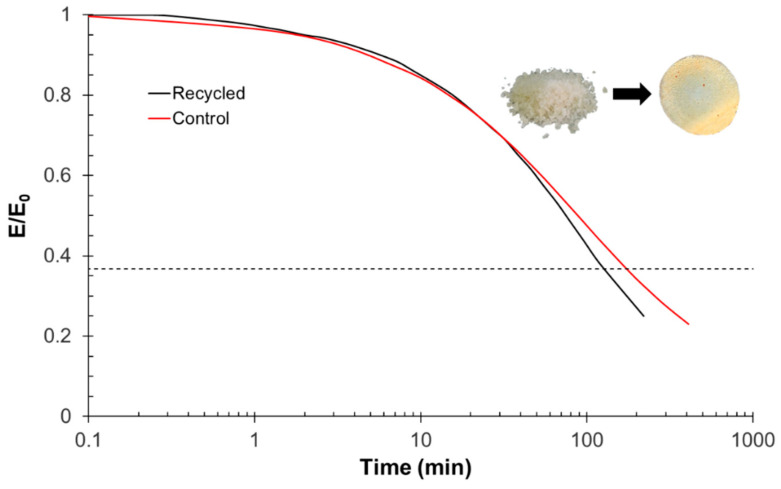
Stress relaxation of recycled FEMA 3D 25 in comparison to its pristine counterpart. Characteristic relaxation time (EE0=1/e) is marked with a dashed line. Inset: Ground sample before recycling (**left**); recycled sample after removal from hot press (**right**).

Interestingly, a damaged material which did not receive post-treatment was indeed fully reparable, as can be seen in Figure 15B. This is probably due to the fact that this material had more freedom to rearrange the topology of its network, since the transesterification equilibrium was not yet achieved solely after treatment at 120 °C. Different reparation schemes were employed for this set of materials. First of all, one sample was repaired by thermally curing at 120 °C, a temperature well below its topology freezing temperature T_v_. The calculation of this parameter is explained in the supporting information. No transesterification reaction is expected to take place at this temperature, and therefore, no repair would be possible other than physical adhesion at the annular interface between the newly cured material and the solid sample. As can be seen, the increase in Young modulus over the damaged sample is limited, in this case (Figure 15B, green line). On the other hand, when the repaired sample is post-treated at 180 °C for 4 h, the reparation was complete, as indicated by a Young modulus practically equal to that of the pristine sample. This shows that transesterifications play a crucial role in establishing a covalent linkage across the repair interface. Besides their reparability, the materials can also be ground into fine particles and recast using pressure and temperature (Figure 16, inset). A rectangular prismatic sample recycled in this way exhibited a full recovery of Young’s modulus (Figure 15B, pink line). Its stress relaxation profile was also identical to that of the pristine sample, as can be seen in Figure 16.

Further mechanical tests were conducted on the FEMA 3D 25 formulation to shed light on its reparability and reprocessability. Standard dog-bone test specimens (type IV, ASTM-D638-14 standard) were used, and three treatments were considered: pristine, assembled (from two toothed halves, see Figure S4 in supporting information), and repaired (after cutting a pristine sample in half). The assembly was performed by applying the liquid formulation at the interface of two halves and applying pressure to ensure good contact, followed by local irradiation using a UV lamp (see Materials and Methods for lamp specifications). To repair the broken halves (i.e., to obtain the “repaired” specimens), a 1–2 mm space was left between the two halves, which was then filled with the liquid formulation to ensure seamless contact. Irradiation was continued, and liquid formulation was added as needed until the union between the two halves was visually defect-free. This was followed by further UV curing in an Asiga UV oven, with a 12 h thermal treatment at 120 °C, followed by a 4 h treatment at 180 °C.

**Figure 17:**
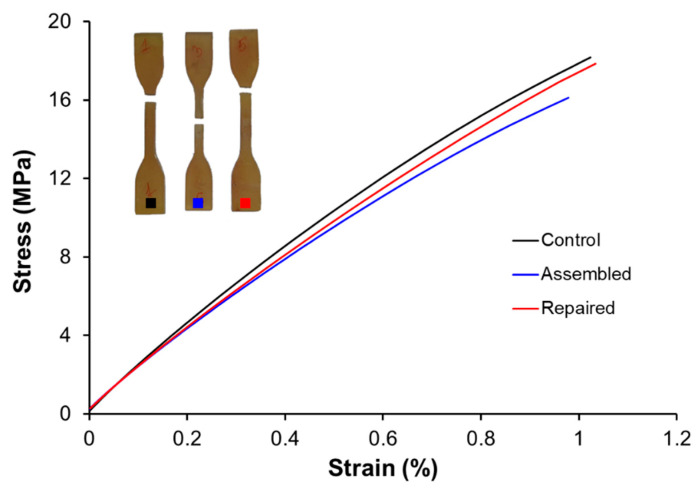
Stress–strain curves of FEMA 3D 25 dog-bone samples. Inset: Photographs of samples after failure.

As can be seen in Table 6 and Figure 17, the pristine (control) specimens have Young’s moduli of about 1700 MPa, a value similar to what was found in the 3-point bending tests. The repaired specimens exhibit comparable Young’s moduli, whereas the assembled specimens show slightly lower values, around 1600 MPa.

As can be seen, while both pristine specimens and the repaired specimen broke at the neck, the assembled specimens broke at the union (Figure 17, inset). This suggests that while the success of the simple repair procedure explained previously was confirmed, the toothed interface might have introduced imperfections that caused local stresses during straining, leading to failure. Nevertheless, the fracture stresses seem to be independent of the specimen type.

## 4. Conclusions

Photoresins with vitrimeric properties for 3D printing exhibit great eligibility in modern manufacturing industries that are driven by customization and make-to order type practices. Considering the ever increasing global need to reduce plastic waste, the ability of these materials to be reprocessed puts them ahead of the competition. We showed how the thermal, thermomechanical, and vitrimeric properties of a hybrid acrylate-epoxy material is impacted by changes in its crosslinking density and the concentration of its dynamic bonds, which are in turn affected by the monomer composition. As the overall functionality of the monomer mixture is increased, such as when there is more trifunctional coupling agent, the crosslinking density is increased. A tighter network, in turn, relaxes its stress more slowly. A similar effect is observed when epoxy monomers with more rigid structures are employed. In light of these findings, we developed a new and improved resin with desirable viscosity for DLP-3D printing, as well as good thermal, mechanical, and vitrimeric performance. The materials printed with this resin could be repaired and recycled using straightforward procedures, with full recovery of their tensile properties.

## Figures and Tables

**Table 1:** Formulations with varied acrylate:epoxy ratios.

Formn.	GLYDA		PEGMA		GMA		DG		GLU	
	g	eq·10^3^	g	eq·10^3^	g	eq·10^3^	g	eq·10^3^	g	eq·10^3^
0.25	0.25	1.44	0.25	0.83	0.816	5.74	2	10.7	1.086	16.4
0.5	0.5	2.87	0.5	1.66	0.816	5.74	2	10.7	1.086	16.4
1 *	1	5.74	1	3.33	0.816	5.74	2	10.7	1.086	16.4
2	2	11.5	2	6.66	0.816	5.74	2	10.7	1.086	16.4
3	3	17.2	3	10	0.816	5.74	2	10.7	1.086	16.4
4	4	23	4	13.3	0.816	5.74	2	10.7	1.086	16.4

* BASE 3D.

**Table 2:** Formulations to test the effect of GMA.

Formn.	GLYDA		PEGMA		GMA		DG		GLU	
	g	eq·10^3^	g	eq·10^3^	g	eq·10^3^	g	eq·10^3^	g	eq·10^3^
0.05	1	5.74	1	3.33	0.056	0.394	3	16	1.086	16.4
0.8 *	1	5.74	1	3.33	0.816	5.74	2	10.7	1.086	16.4
2.4	1	5.74	1	3.33	2.34	16.4	0	0	1.086	16.4

* BASE 3D.

**Table 3:** Formulations containing different epoxides.

Formn.	GLYDA		PEGMA		GMA		Epoxy		GLU	
	g	eq·10^3^	g	eq·10^3^	g	eq·10^3^	g	eq·10^3^	g	eq·10^3^
NG	1	5.74	1	3.33	0.816	5.74	1.28	10.7	1.086	16.4
CE	1	5.74	1	3.33	0.816	5.74	2.14	10.7	1.086	16.4
TGE	1	5.74	1	3.33	0.816	5.74	1.49	10.7	1.086	16.4
PPGGE	1	5.74	1	3.33	0.816	5.74	3.42	10.7	1.086	16.4
PPGGEs	1	5.74	1	3.33	0.816	5.74	2.03	10.7	1.086	16.4
TFG	1	5.74	1	3.33	0.816	5.74	1.29	10.7	1.086	16.4
DG *	1	5.74	1	3.33	0.816	5.74	2	10.7	1.086	16.4

* BASE 3D.

**Table 4:** T_g_ (in °C) of cured materials at 120 °C before and after post-treatment of 4 h at 180°C, as influenced by epoxide type.

Post-Treatment	NG	CE	TGE	PPGGE	PPGGEs	TFG	DG
Before	−18	8	8	−45	−5	38	42
After	4	32	24	−42	−3	43	47

**Table 5:** 3-D printing formulations.

Formn.	GMA		PPGGEc		DG		GLU	
	g	eq·10^3^	g	eq·10^3^	g	eq·10^3^	g	eq·10^3^
FEMA 3D 25 *	0.816	5.74	0.5	2.67	1.5	8.02	1.086	16.4
FEMA 3D 50	0.816	5.74	1	5.35	1	5.35	1.086	16.4
BASE 3D	0.816	5.74	0	0	2	10.7	1.086	16.4

* Selected formulation for reprocessing tests; viscosity: 0.47 Pa·s.

**Table 6:** Average tensile test results of different treatments of FEMA 3D 25 samples.

Specimens	Control	Assembled	Repaired
Young’s modulus (MPa)	1741	1628	1710
Fracture stress (MPa)	17.9	15.9	17.0

## Data Availability

The raw data used in this work is available upon request from the authors.

## Supplementary Materials

The following supporting information can be downloaded at: https://www.mdpi.com/article/10.3390/polym14235102/s1, Figure S1: FTIR absorbance spectra of acrylate/methacrylate C=C bonds (1) and carbonyls (2) during UV cure at ambient temperature. Elapsed time between initial and final spectra: 40 s; Figure S2: FTIR absorbance spectra of epoxy bonds during epoxy-acid reaction at 120°C. The absorbance peak disappears in a little over 2 h; Figure S3: Normalized relaxation modulus of FEMA 3D 25 at different temperatures. The dashed line marks *E/E_0_ = 1/e*, which corresponds to the characteristic relaxation time $\tau^{*}$. Inset: Arrhenius plot used to compute the activation energy of relaxation $E_{a}$; Table S1: Kinetic parameters of stress relaxation of FEMA 3D 25. The value of $E^{\prime }$ used in the calculations is also shown; Figure S4: Toothed halves printed using FEMA 3D 25 to be assembled and tensile tested.
