# Supplementary material for: Optimization and Testing of Hybrid 3D Printing Vitrimer Resins
Source: Polymers (Basel). 2022 Nov 24;14(23):5102. doi: 10.3390/polym14235102 (PMC9739315; doi:10.3390/polym14235102)
Supplement: Supplementary file 1 [file polymers-14-05102-s001.zip › polymers-2043039-SI.pdf]

SUPPORTING INFORMATION

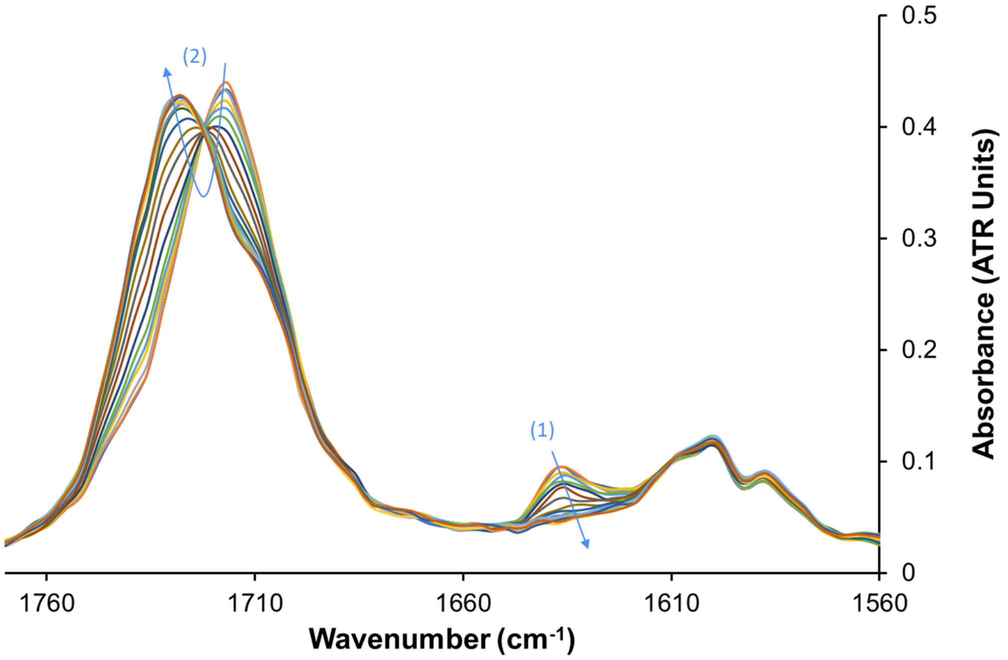

Figure S1. FTIR absorbance spectra of acrylate/methacrylate C=C bonds (1) and carbonyls (2) during UV cure at ambient temperature. Elapsed time between initial and final spectra: 40 s

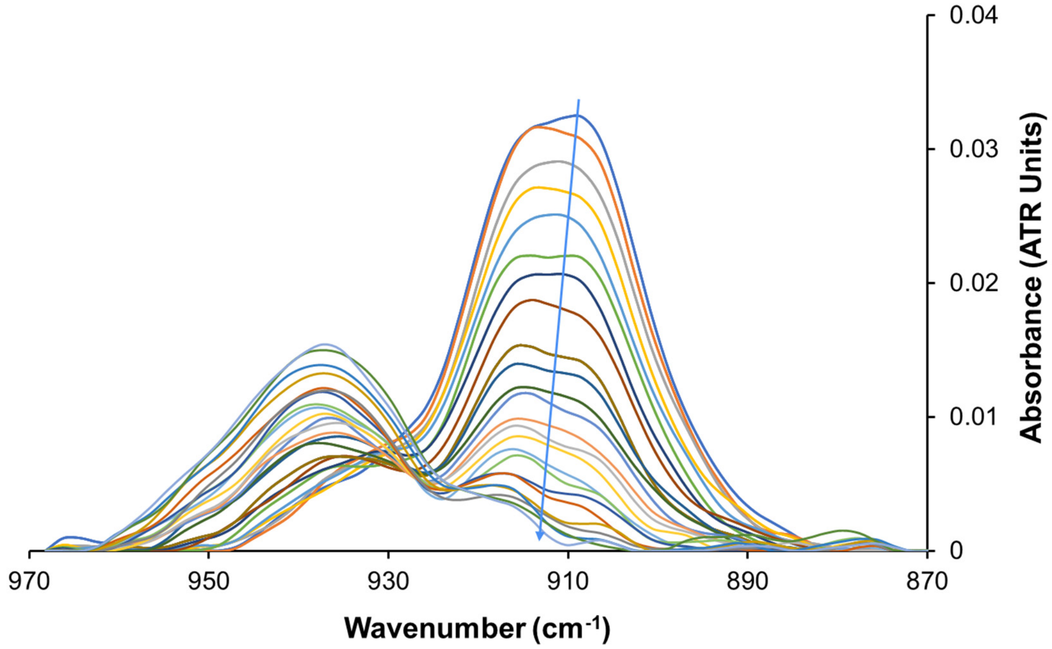

Figure S2. FTIR absorbance spectra of epoxy bonds during epoxy-acid reaction at 120ºC. The absorbance peak disappears in a little over 2 h.

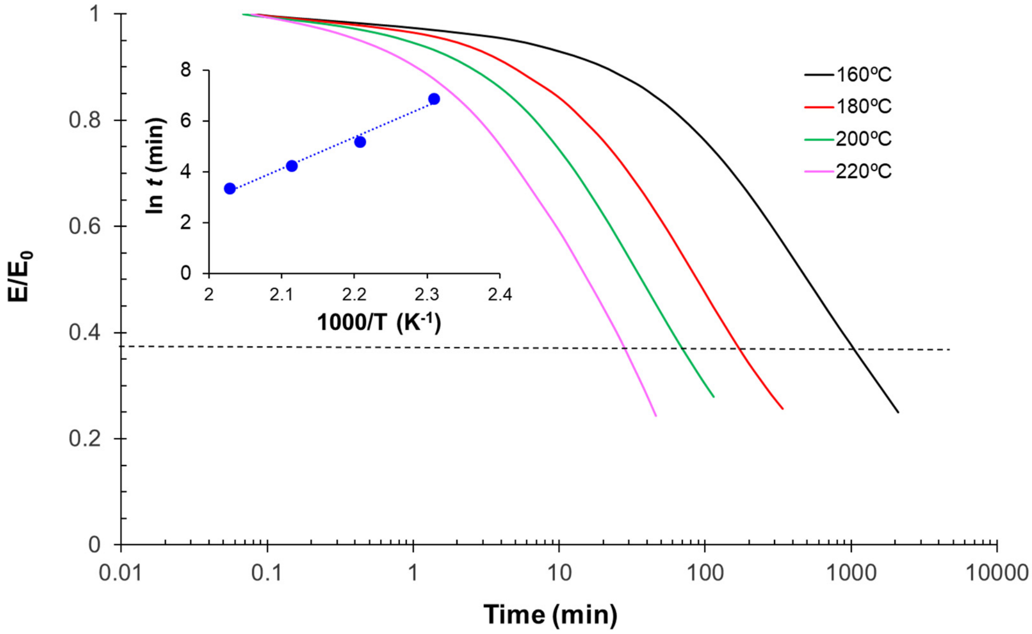

Figure S3. Normalized relaxation modulus of FEMA 3D 25 at different temperatures. The dashed line marks *E/E₀=1/e*, which corresponds to the characteristic relaxation time τ^*. Inset: Arrhenius plot used to compute the activation energy of relaxation E_a

In vitrimers and vitrimer-like materials, characteristic relaxation times τ^* (i.e. time required to relax ~63% of the stress) exhibit Arrhenius-like behavior represented mathematically by $\tau(T) = \tau_0 \cdot \exp(E_{act}/RT)$, and plotted as in Figure S3 (inset) for FEMA 3D 25. The topology freezing temperature T_v, below which the transesterification reactions are assumed negligibly slow, was determined following the procedure by Capelot et al (ACS Macro Lett. 1, 2012, pp. 789-792). It is assumed that liquid-to-solid transition occurs (i.e. the topology freezes) once the viscosity exceeds10^{12} Pa.s. Using the Maxwell equation $\eta = G \cdot \tau^*$, the characteristic time that corresponds to this viscosity can be calculated. The shear modulus and tensile modulus are related through the equation $G = E'/[2(1+\nu)]$ where ν is the Poisson's ratio (assumed equal to 0.5 in the rubbery state). Using E' determined by DMA, T_v can be calculated in a straightforward manner.

Table S1. Kinetic parameters of stress relaxation of FEMA 3D 25. Also shown is the value of E' used in the calculations

E' (MPa)	E_a (kJ/mol)	ln(τ_0) (min)	T_v (ºC)
17	103	22	157

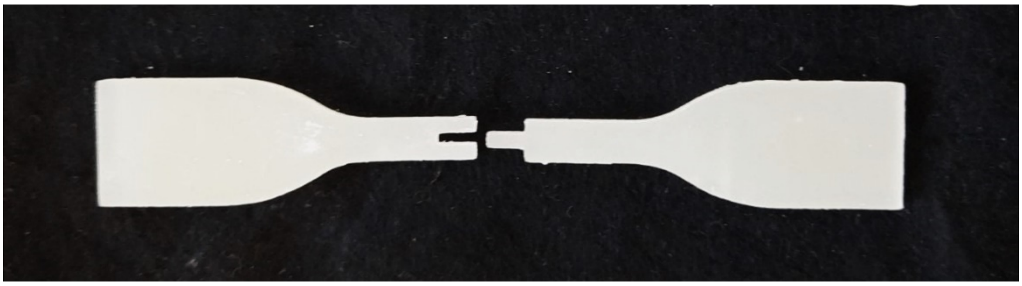

Figure S4. Toothed halves printed using FEMA 3D 25 to be assembled and tensile tested.
